# ^99m^Tc-labelled anti-CD11b SPECT/CT imaging allows detection of plaque destabilization tightly linked to inflammation

**DOI:** 10.1038/srep20900

**Published:** 2016-02-15

**Authors:** Guobing Liu, Yan Hu, Jie Xiao, Xiao Li, Yanli Li, Hui Tan, Yanzhao Zhao, Dengfeng Cheng, Hongcheng Shi

**Affiliations:** 1 Department of Nuclear Medicine, Zhongshan Hospital, Fudan University, Shanghai 200032, China; 2 Institute of Nuclear Medicine, Fudan University, Shanghai 200032, China; 3 Shanghai Institute of Medical Imaging, Shanghai 200032, China

## Abstract

It remains challenging to predict the risk of rupture for a specific atherosclerotic plaque timely, a thrombotic trigger tightly linked to inflammation. CD11b, is a biomarker abundant on inflammatory cells, not restricted to monocytes/macrophages. In this study, we fabricated a probe named as ^99m^Tc-MAG_3_-anti-CD11b for detecting inflamed atherosclerotic plaques with single photon emission computed tomography/computed tomography (SPECT/CT). The ApoE-knockout (ApoE^−/−^) mice were selected to establish animal models, with C57BL/6J mice used for control. A higher CD11b^+^-cell recruitment with higher CD11b expression and more serious whole-body inflammatory status were identified in ApoE^−/−^ mice. The probe showed high *in vitro* affinity and specificity to the Raw-264.7 macrophages, as well as inflammatory cells infiltrated in atherosclerotic plaques, either in *ex vivo* fluorescent imaging or in *in vivo* micro-SPECT/CT imaging, which were confirmed by *ex vivo* planar gamma imaging, Oil-Red-O staining and CD11b-immunohistochemistry staining. A significant positive relationship was identified between the radioactivity intensity on SPECT/CT images and the CD11b expression in plaques. In summary, this study demonstrates the feasibility of anti-CD11b antibody mediated noninvasive SPECT/CT imaging of inflammatory leukocytes in murine atherosclerotic plaques. This imaging strategy can identify inflammation-rich plaques at risk for rupture and evaluate the effectiveness of inflammation-targeted therapies in atheroma.

Cardiovascular disease (CVD) has become the first fatal disease in recent years, with approximately 235.5 out of 100,000 people being affected, contributing to a mortality of about 31.0%^1^. When taking stroke into consideration, the number of people dying of CVD would account for 1/3 of the total deaths^1^. In most cases, CVD is associated with atherosclerotic vascular diseases and their sequelae, e.g., myocardial infarction and stroke. Interestingly, among the factors determining the atherosclerosis-associated acute cardiovascular events, the composition of plaques is far more important than arterial stenosis, with plaque rupture and the secondary thromboembolism being the primary etiology^2^. Patients with acute ischemic events usually harbor multiple ruptured atherosclerotic plaques^3^,^4^. Thus, early identification of unstable plaque before rupture is the current challenge facing clinical physicians.

Inflammatory cells play an important role in pathogenesis of atherosclerosis and its complications. Plaque at risk of rupture is characterized by high infiltration of inflammatory cells. Much of the mayhem caused by inflammatory cells inside lesions is inflicted through secreting cytokines, reactive oxygen species, and proteolytic enzymes which degrade extracellular matrix and weaken the fibrous cap that protects the plaque against rupture^5^,^6^. Therefore, inflammatory cells furnish attractive imaging biomarkers for distinguishing the atherosclerotic lesions from benign to vulnerable plaques^5^. Targeting these cells with imaging may offer early insight into the course of disease.

In recent years, some relevant strategies targeting plaque inflammation were proposed via nuclear medical techniques. For example, ^125^I/^99m^Tc-MCP-1 and ^99m^Tc-B2702 (VCAM-1) target for inflammation-associated factors, utilizing highly expressed receptor or specific immunity^5^,^7^,^8^. More specifically, ^111^In-monocytes and ^11^C/^3^H-PK11195 targeting peripheral benzodiazepine receptor on macrophages that abundantly exist in the vulnerable plaques^9^,^10^, as well as ^99m^Tc-Annexin V targeting macrophage apoptosis were developed^11^,^12^,^13^. However, inflammatory cells infiltrated in plaques are not limited to monocytes/macrophages. It is reported that, about 11.8%, 0.7% and 10.8% of the total cells infiltrated in atherosclerotic plaques were neutrophils, lymphocytes and other inflammatory cells, respectively, other than monocytes/macrophages^14^. Therefore, those probes may hinder timely inflammation imaging due to limited sensitivity. Seemingly, the ^18^F-FDG/^11^C-Cholin PET can image the inflammation, but with relatively low specificity^15^,^16^. Besides, recent studies have suggested that FDG accumulation may reflect hypoxia-stimulated macrophages rather than inflammation per se^17^.

In contrast, CD11b (also known as Mac-1), an active constituent of the innate immune response predominately expressed on monocytes/macrophages, granulocytes, myeloid-derived dendritic cells, natural killer cells, microglia, B-1 cells and activated neutrophils, is a more sensitive biomarker of inflammatory cells, which has been reported overexpressed in tumor tissues^18^, infarcted myocardium, and atherosclerosis plaques^19^,^20^. Targeting these cells may reflect an interesting approach to further enhance the non-invasive detection of rupture-prone atherosclerosis plaques. However, up to now, there is no *in vivo* nuclear medical imaging approach reported utilizing CD11b-related affinity. In this study, we fabricated a ^99m^Tc-labeled anti-CD11b antibody as a probe for detecting inflamed atherosclerotic plaques with single photon emission computed tomography (SPECT).

## Results

### Probe synthesis, stability, blood half-life, and biodistribution

**Figure 1:**
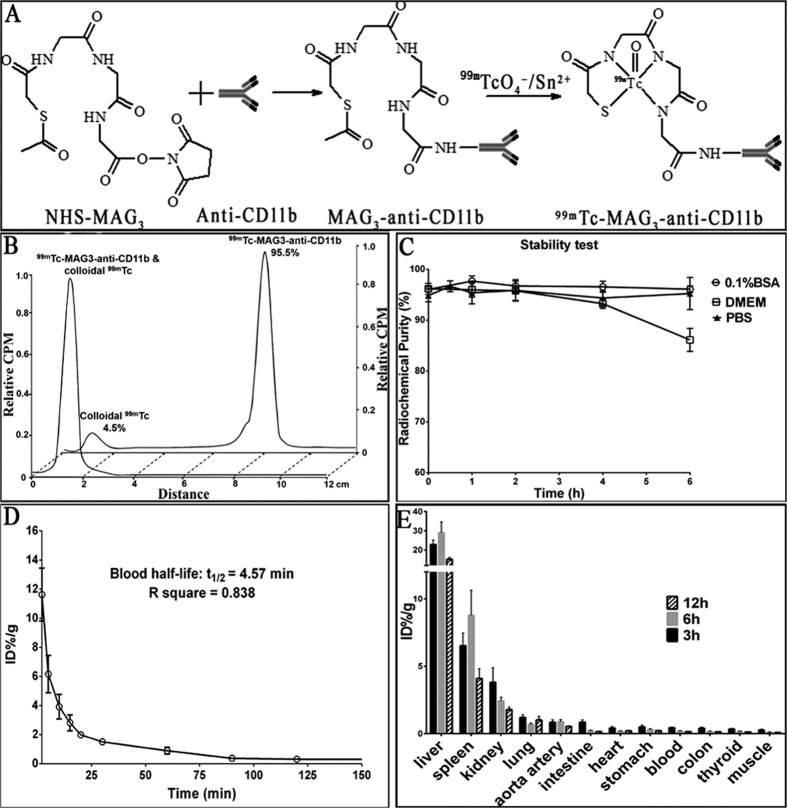
Probe preparation and pharmacokinetics. (**A**) Preparation schema of ^99m^Tc-MAG_3_-anti-CD11b antibody. (**B**) Radio-thin layer chromatography traces for ^99m^Tc-MAG_3_-anti-CD11b and colloidal ^99m^Tc, with the radio-purity of ^99m^Tc-MAG_3_-anti-CD11b measured to be 95.5%. (**C**) *In vitro* stability tests of the probe fabricated in BSA, DMEM and PBS solution, which could be obtained to 6 h after labelling. (**D**) Blood half-life of ^99m^Tc-MAG_3_-anti-CD11b. (**E**) Biodistributions at 3 h, 6 h and 12 h after administration of ^99m^Tc-MAG_3_-anti-CD11b (n = 5 for each time point). CPM indicates counts per minute; BSA, bovine serum albumin; DMEM, dulbecco’s modified eagle’s medium; PBS, phosphate buffered solution; %ID/g, percent injected dose per gram tissue.

The synthesis of ^99m^Tc-labeled anti-CD11b is described in the methods section and is shown in Fig. 1A. The radiolabeling yield of ^99m^Tc-labeled anti-CD11b was over 95% (Fig. 1B) with favorable stabilities in PBS, BSA and DMEM till at least 6 hours post labelling (Fig. 1C). The *in vivo* kinetic test showed the probe had a blood half-life of 4.57 ± 0.59 min (R square = 0.83; Fig. 1D), indicating a quick clearance from the blood pool. Biodistributions of ^99m^Tc-labeled anti-CD11b (expressed as %ID/g) in C57 mice, 3, 6 and 12 hours after injection, were presented in Fig. 1E, which demonstrated a typical *in vivo* distribution of protein. High accumulations of radioactivity could be observed in liver and spleen, both of which went up till 6 h after injection but declined 12 h after injection.

### Probe affinity and specificity to inflammatory cells

**Figure 2:**
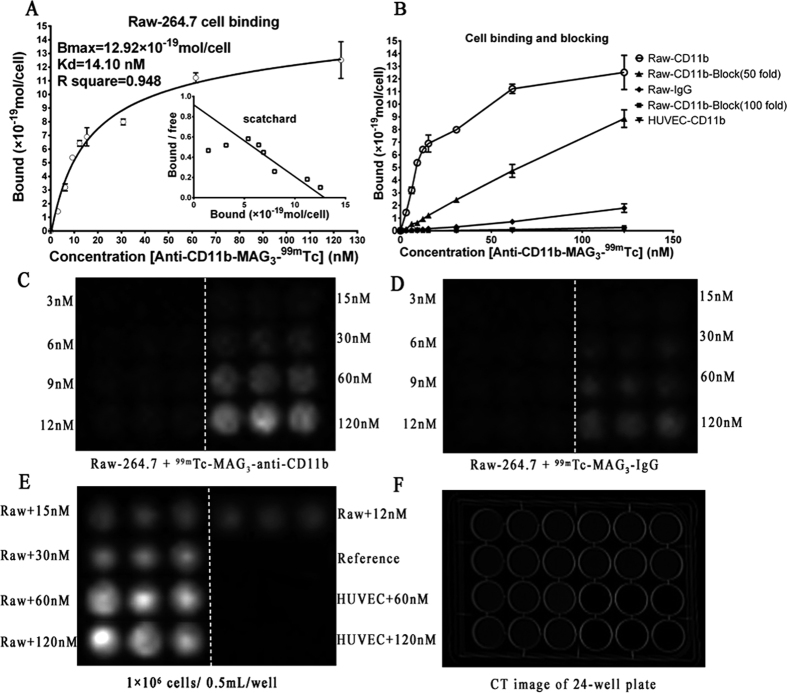
Cellular binding and blocking experiment *in vitro*. (**A**) Saturation binding curves generated by measuring the radioactivity in Raw-264.7 cells incubated with increasing concentrations of ^99m^Tc-MAG_3_-anti-CD11b. The inset is the Scatchard plot from the saturation binding experiment. (**B**) Saturation binding curves by measuring the radioactivity in Raw-264.7 cells incubated with ^99m^Tc-MAG_3_-IgG and by measuring the radioactivity in HUVEC cells incubated with ^99m^Tc-MAG_3_-anti-CD11b, both with same concentrations gradient as (**A**), as well as blocking curves with a dosage of 50-fold and 100-fold unlabeled anti-CD11b antibody blocking receptor on Raw-264.7 cells before saturation binding experiment as done in (**A**). (**C**) Planar imaging by BSGI of the 24-well plate with Raw-264.7 cells incubated with increasing concentrations of ^99m^Tc-MAG_3_-anti-CD11b. (**D**) Planar imaging by BSGI of the 24-well plate HUVEC cells incubated with same concentration gradient of ^99m^Tc-MAG_3_-anti-CD11b. (**E**) Planar imaging by BSGI of the 24-well plate with an assemble of Raw-264.7 cells and HUVEC cells incubated with ^99m^Tc-MAG_3_-anti-CD11b of different concentrations. (**F**) CT imaging of the 24-well plate. White dashed line in (**C–E**) indicate experiments were triplicated on both side.

To assess the cellular affinity of ^99m^Tc-labeled anti-CD11b antibody, saturation binding experiments were performed using the Raw-264.7 cell line. By incubating Raw-264.7 cells with increasing concentrations of ^99m^Tc-labeled anti-CD11b antibody, the K_D_ value was found to be 14.10 ± 5.06 nM and the Bmax value was 1.29 ± 0.23 × 10^5^ receptors/cell (R square = 0.948, Fig. 2A). Taking the nonspecific IgG as targeting antibody instead of anti-CD11b antibody, we found that the probe affinity to Raw-264.7 were significantly higher than the nonspecific IgG binding to cells at each concentration point (Fig. 2B). In contrast, the human umbilical vein endothelial cell (HUVEC) showed almost none affinity to the probe fabricated (Fig. 2B). To further confirm the specific binding of the probe to Raw-264.7 cell line, we performed the blocking studies with a 50-folds and a 100-folds unlabeled anti-CD11b antibody with same concentration gradient as used in saturation test to block the CD11b on membrane of Raw-264.7 cells, which showed an incomplete blocking by the former dosage and a complete blocking by the latter (Fig. 2B). Statistical analysis revealed that there were significant differences in cell binding between the unblocked and blocked groups at all the tested concentration points under both blocking dosage. The images captured by breast specific gamma imaging (BSGI, Dilon 6800, Dilon Technologies, Newport News, VA) of the 24-well plates after the cell binding and blocking tests offered overall visions of the probe affinity and specificity as shown in Fig. 2C–E. These data suggest that ^99m^Tc-labeled anti-CD11b antibody specifically binds to Raw-264.7 cells, which constitute the majority of inflammatory cells.

### Establishment of animal models and evaluations

**Figure 3:**
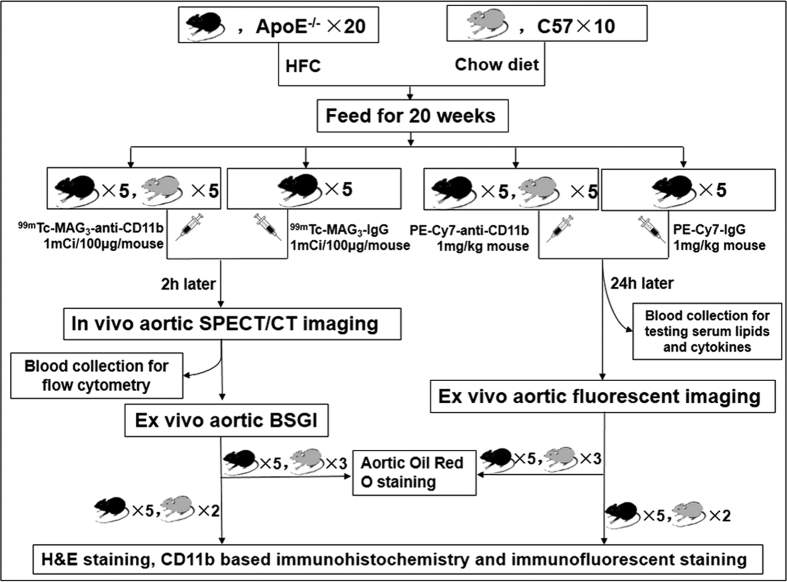
Flow chart of animal experiments.

**Figure 4:**
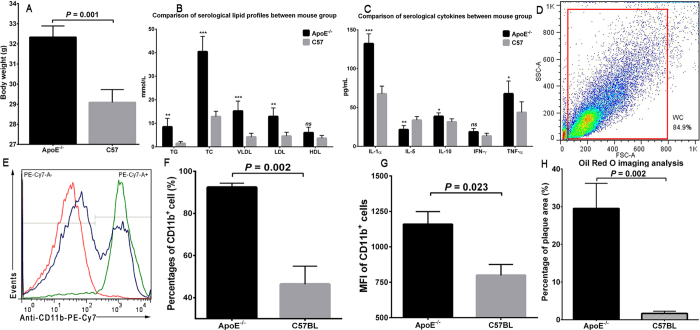
Data supporting successful establishment of animal models of atherosclerosis. (**A**) Comparison of the body weight when fed for 20 weeks between the ApoE^−/−^ and C57 mice. (**B,C**) Comparison of serological lipid profiles and cytokines between these two mouse groups. “*”, “**” and “***” indicate P < 0.05, <0.01 and <0.001, respectively; while “ns” denotes not significant. (**D**) Flow cytometry of circulating blood. The plot in red color denotes gate for blood white cells. (**E**) Signal curves that report signal intensity in PE-Cy7 FACS channel, which represent anti-CD11b-PE-Cy7 binding to CD11b^+^cells. The green curve indicates a sample from ApoE^−/−^ mouse, the blue curve indicates a sample from C57 mice, while the red curve is isotype for control. (**F**) Bar graph illustrates percentage-difference of CD11b^+^cells between mouse groups. (**G**) Bar graph illustrating difference of mean fluorescent intensity in CD11b^+^cells between mouse groups. MFI denotes mean fluorescent intensity. (**H**) Image analysis of Oil Red O staining showing higher percentage of plaque area in ApoE^−/−^ mice than in C57 mice.

Experiments refer to animals were illustrated in Fig. 3. The ApoE^−/−^ mice were significantly heavier than the C57 mice (32.3 ± 0.56 g *vs*. 29.1 ± 0.64 g; *P* = 0.001) at the time of experiments after 20 weeks feeding, as shown in Fig. 4A. The ApoE^−/−^ mice also showed higher concentrations of serological triglyceride (TG), total cholesterol (TC), low density lipoprotein (LDL) and very low density lipoprotein (VLDL), with *P* values being all less than 0.05, as shown in Fig. 4B, while inter-group difference of high density lipoprotein (HDL) was not significant (*P* = 0.078). Furthermore, serological IL-1α, IL-10, and TNF-α were identified higher in ApoE^−/−^ mice (*P* values all less than 0.05; Fig. 4C), whereas IL-5 was significantly lower in ApoE^−/−^ mice (*P* < 0.01; Fig. 4C). Inter-group discrepancy of serological IFN-γ was not significant (*P* = 0.066; Fig. 4C). The percentage of circulating blood CD11b^+^ cells was also significantly larger in ApoE^−/−^ mice (92.4 ± 2.01%) than in C57 mice (46.4 ± 8.60%) demonstrated by flow cytometry (*P* = 0.002; Fig. 4D–F). Besides, the CD11b^+^ cells from ApoE^−/−^ mice presented larger mean fluorescent intensity (MFI,) than those from C57 mice (MFI, 1159 ± 90.69 *vs*. 799 ± 76.89, *P* = 0.023; Fig. 4G), which implied higher CD11b expression of CD11b^+^ cells in ApoE^−/−^ mice. All these indicated a whole-body inflammatory status in ApoE^−/−^ mice and a higher mobilization of inflammatory cells to circulation system. Next, we compared the percentage area of plaques to the total area of aortas between ApoE^−/−^ mice and C57 mice based on Oil Red O staining. The ApoE^−/−^ mice presented significantly larger areas of aortic atherosclerosis than C57 mice (29.5 ± 6.67% *vs*. 1.7 ± 0.61%, *P* = 0.002; Fig. 4H). The above results indicated successful model establishment of atherosclerosis in ApoE^−/−^ mice and thereby assuring further evaluation.

### *Ex vivo* aortic fluorescent imaging proved probe targeting ability

**Figure 5:**
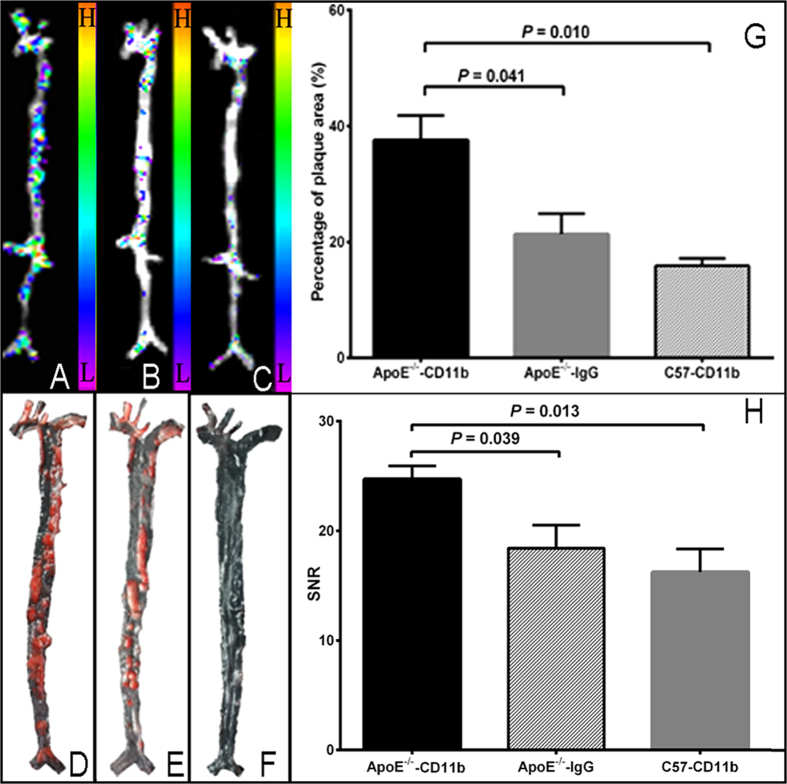
*Ex vivo* aortic fluorescent imaging and corresponding imaging analysis. Fluorescent imaging of aortas from ApoE^−/−^ mice (**A**,**B**) and C57 mice (**C**) 24 h after injection of ani-CD11b-PE-Cy7 (**A,C**) and IgG-PE-Cy7 (**B**). “H” means high signal, and “L” means low signal (similarly hereinafter). (**D–F**) Images of Oil Red O staining of aortas corresponding to (**A–C**) orderly. (**G,H**) Respectively **c**omparison of plaque-area percentages and signal/noise ratio (SNR) between groups, respectively. The *P*-values presented were adjusted *P* values from Tukey’s multiple comparisons test.

*Ex vivo* near-infrared fluorescence imaging (NIRFI) was performed on excised aortas 24 h post injection. As compared to the C57 mice, the plaque-area percentages identified by anti-CD11b NIRFI were significantly higher in aortas of ApoE^−/−^ mice (37.6 ± 4.27% *vs*. 15.9 ± 1.31, *P* = 0.010; Fig. 5A,C,G). Within the ApoE^−/−^ mice group, the plaque-area demonstrated by anti-CD11b targeting NIRFI was significantly larger than nonspecific IgG targeting NIRFI (percentages: 37.6 ± 4.27% *vs*. 21.3 ± 3.53, *P* = 0.041; Fig. 5A,B,G). Furthermore, the aortas from ApoE^−/−^ mice that received anti-CD11b NIRFI displayed strong fluorescence signal, which were predominantly distributed in areas where extensive atherosclerotic lesions were expected as described by Nakashima *et al.*^21^. Interestingly, the signal-to-noise ratio (SNR) in anti-CD11b targeting NIRFI of ApoE^−/−^ mice aortas (24.7 ± 1.207) was significantly higher than in anti-CD11b targeting NIRFI of C57 mice aortas (16.2 ± 2.10), and than in IgG targeting NIRFI of ApoE^−/−^ mice aortas (18.4 ± 2.108), with *P*-values being 0.013 and 0.039, respectively (Fig. 5A–C,H). The fluorescence intensities and distributions of these aortas were consistent with the Oil Red O staining (Fig. 5A–F). These indicate the affinity and specificity of anti-CD11b antibody to the inflammatory environment of atherosclerotic plaque can be obtained in *ex vivo* tissue condition.

### *In vivo* Micro-SPECT/CT Imaging displayed plaque inflammation

**Figure 6:**
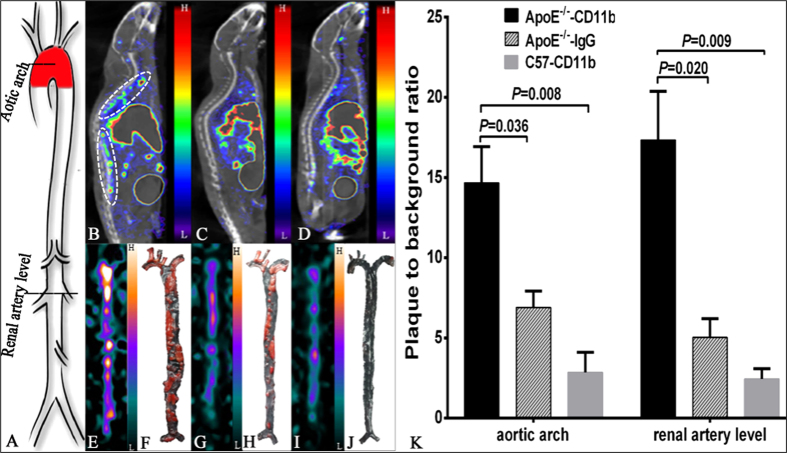
Data analysis and validation of *in vivo* SPECT/CT imaging. (**A**) Schematic diaphragm of the whole aorta illustrates positons selected for semi-quantitative analysis. Sagittal SPECT/CT images of ApoE^−/−^ mice (**B**,**C**) and a C57 mouse (**D**) scanned at 2 h after injection of ^99m^Tc-MAG_3_-ani-CD11b (**B**,**D**) and ^99m^Tc-MAG_3_-IgG (**C**). (**E,G**,**I**) were, in order, the corresponding *ex vivo* BSGI planar images of the aortas excised from the mice in (**B**–**D**) right after SPECT/CT imaging, while the Oil Red O staining of these aortas were presented in (**F**,**H**,**J**), orderly. (**K**) Comparison of lesions’ radioactivity on SPECT/CT expressed as plaque-to-background ratio between groups. The *P*-values presented were adjusted *P* values from Tukey’s multiple comparisons test. White circles in (**B)** denote atherosclerotic plaques with obvious accumulation of radioactivity.

^99m^Tc-MAG_3_-anti-CD11b uptake in atherosclerotic plaques at levels of aortic arch and at branch point of renal artery were semi-quantitatively assessed with micro SPECT/CT as described in method section (Fig. 6A). The ApoE^−/−^ mice had significantly higher radioactivity, expressed as plaque to background ratio (P/B), than the C57 mice both at aortic arch (P/B: 14.7 ± 2.26 versus 2.9 ± 0.72, *P* = 0.008; Fig. 6B,D,K) and at levels of renal artery (P/B: 17.3 ± 3.04 versus 2.5 ± 0.36, *P* = 0.009; Fig. 6B,D,K). Furthermore, within the ApoE^−/−^ mice group, the ^99m^Tc-MAG_3_-anti-CD11b based imaging also showed significant higher P/B ratios than the ^99m^Tc-MAG_3_-IgG based imaging both at aortic arch (P/B: 14.7 ± 2.26 versus 6.9 ± 0.60; *P* = 0.036; Fig. 6B,C,K) and at levels of renal artery (P/B: 17.3 ± 3.04 versus 5.0 ± 0.68, *P* = 0.020; Fig. 6B,C,K). These indicated a good *in vivo* targeting of anti-CD11b antibody to inflammatory environment of plaques.

### *Ex vivo* aortic planar imaging, Oil Red O staining and immunohistochemistry confirmed *in vivo* SPECT/CT imaging

After micro-SPECT/CT imaging, we performed planar imaging of the excised aortas through BSGI. In planar images, radioactivities distributed along the whole aortas of ApoE^−/−^ mice but most intensively in aortic arches and abdominal aortas (Fig. 6E). Interestingly, relative lower accumulations of radioactivities were observed in ^99m^Tc-MAG_3_-IgG based planar aortic images of ApoE^−/−^ mice (Fig. 6G) and in ^99m^Tc-MAG_3_-anti-CD11b based planar aortic images of C57 mice (Fig. 6I). The distributions of radioactivities in aortas presented by planar imaging were consistent with the distributions reflected by micro-SPECT/CT (Fig. 6B–D). Furthermore, Oil Red O staining of the excised aortas in current study totally reproduced the SPECT/CT and BSGI images, as shown in Fig. 6F,H,J.

**Figure 7:**
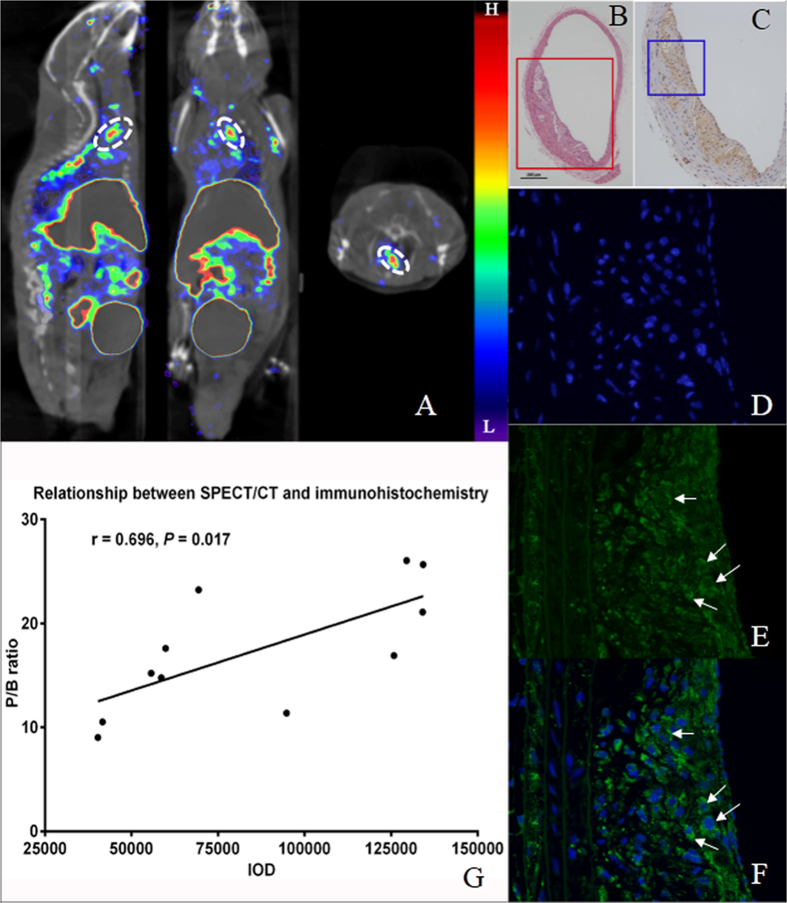
Pathological and immunohistochemical confirmation of *in vivo* SPECT/CT imaging. (**A**) ^99m^Tc-MAG_3_-anti-CD11b SPECT/CT images (sagittal, coronal, and transverse views, left to right) of an ApoE^−/−^ mouse, with radioactivities obviously accumulated in aortic arch, thoracic aorta and abdominal aorta, which distributed along the spinal column. (**B**) Hematoxylin and eosin staining (×40) of tissue section sliced at position marked in white circles in (**A**). (**C**) CD11b immunohistochemical image (×100) selected at area corresponding to position marked in red rectangle in (**B)**. (**D–F**) Higher magnification (×400) immunofluorescence microscopy of DAPI staining (by which nucleus were stained blue), CD11b staining (stained in green) and the merged image, selected in area corresponding to blue rectangle in (**C**). Arrows illustrate CD11b expression mainly located around the nucleus, indicating a cellular-surface expression of CD11b. (**G**) Spearman correlation analysis between plaque radioactivity (expressed as P/B ratio) and tissue-CD11b expression in corresponding area measured by immunohistochemistry. IOD denotes integrated optic density.

In order to investigate infiltration of inflammatory cells within the plaques, hematoxylin-eosin (HE) staining, anti-CD11b based immunohistochemistry staining and immunofluorescent staining were performed to aortic sections sliced at points with obvious radioactivities in SPECT/CT images, commonly at level of aortic arch and at branch point of renal artery. The lesions with accumulated radioactivities were consistent with the anatomical structure of the plaques (Fig. 7A,B) and the large amount of CD11b^+^ inflammatory cells infiltrated, as well as the high CD11b expression on inflammatory cells (Fig. 7C–F). The immunofluorescence staining indicated that the CD11b mainly expressed on cellular membrane as they mainly located surrounding the nucleus which were stained blue (Fig. 7D–F). Interestingly, lesions with high radioactivities normally correspond to high expression of CD11b, expressed as integrated optic density (IOD) measured by Image Pro Plus, as described in the method section. Spearman correlation analysis clarified the relationship between the lesions’ activity on SPECT/CT and expression of CD11b on immunohistochemistry staining (r = 0.696, *P* = 0.017; Fig. 7G).

## Discussion

Up till now, it remains challenging to predict the risk of rupture for a specific atherosclerotic plaque, a thrombotic trigger tightly linked to inflammation. Identification of inflamed atheroma could trigger aggressive risk factor modification, intensive pharmacological treatment, and perhaps other preemptive interventions to enhance lesion stability and reduce the probability of plaque rupture and thrombosis. The recruitment of inflammatory cells from the circulating blood to the intima of atherosclerotic vessel walls is a pivotal step in atherogenesis^22^. Matrix-degrading proteases, cytokines, reactive oxygen species tightly linked to infiltration of inflammatory cells, and implicated in plaque rupture. Thus, imaging inflammatory cells will provide a unique tool to identify atherosclerotic plaques that are prone to rupture, and allow prophylactic treatment for plaques before rupture, clotting and occlusion of vessels such as in myocardial infarction^5^.

In this study, we fabricated a nuclear medical imaging probe named as ^99m^Tc-MAG_3_-anti-CD11b to identify inflammatory status in atherosclerotic plaques, taking CD11b (Mac-1) as the central mediator, which predominately expressed in myeloid-derived suppressor cells or immature myeloid cells, specifically corresponding to inflammatory microenvironment^19^,^20^. Compared to the isotype-IgG mediated agent, this probe showed high *in vitro* affinity to the Raw-264.7 macrophage cell lines, which constitute the majority of inflammatory cells, rather than the CD11b negative HUVEC cells. Blocking the Mac-1 receptor with unlabeled anti-CD11b antibody resulted in a significant decrease of probe binding to Mac-1-expressing cells. These findings are consistent with study conducted by von zur Muhlen *et al.*^23^, which identified an increased cell binding and uptake of superparamagnetic iron oxide (SPIO) nanoparticles in Mac-1 activated monocytes/macrophages. The author advocated that the versatile integrin Mac-1 (CD11b) was involved in leukocyte adhesion, complement activation and phagocytosis. Thus, Mac-1 may be useful vehicles for molecular labeling to inflammatory cells of atherosclerotic plaques.

The effective and specific affinity between the anti-CD11b and inflammatory cells infiltrated in atherosclerotic plaques was also obtained in *ex vivo* aortic anti-CD11b targeting fluorescence imaging and *in vivo* anti-CD11b targeting micro-SPECT/CT imaging, which were confirmed by *ex vivo* BSGI planar imaging, Oil Red O staining and immunohistochemistry staining in current study. The planar view of the radioactivity distribution is a direct reflection of atherosclerotic plaques and more closely corresponds to vulnerability. Furthermore, by use of planar imaging, the signal-to-noise ratio could be enhanced to accurately recognize small foci with low uptake, avoiding interference from the surrounding tissue that with high radioactivity accumulation. Poor clearance of inflammatory cells may lead to the accumulation of cellular debris within the lipid-rich core of atherosclerotic plaque; thus, lipid-related Oil Red O staining reflects the amount of inflammatory cells infiltrated, as well as the vulnerability of atherosclerotic plaques^24^. As for the immunohistochemistry, it is even more reliable to look insight into plaques to see the expression of CD11b, directly. Interestingly, the radioactivity intensity on SPECT/CT images significantly correlated with CD11b expression presented in immunohistochemistry. All these indicated the possibility of ^99m^Tc-MAG_3_-anti-CD11b in diagnosing atherosclerosis and in evaluating severity of inflammation within plaques that tightly linked to the risk of rupture.

The infiltration of CD11b^+^ cells, including monocytes/macrophages, granulocytes, myeloid-derived dendritic cells, natural killer cells, microglia, B-1 cells and activated neutrophils, gives impetus to the initiation and progression of inflammation within atheroma, and thus offers targeting point for early detection of inflamed plaques^19^,^20^,^25^,^26^,^27^. In current study, the higher CD11b^+^ cells of higher CD11b-expression in circulating blood of ApoE^−/−^ mice (compared to the C57 mice) identified by flow cytometry, indicated a higher recruitment of CD11b^+^ cells to circulation system, which indirectly supported the importance of CD11b^+^ cells in the formation and development of atheroma. The high uptake of fabricated probe in spleen as shown either in *in vivo* SPECT/CT imaging or in *ex vivo* organ distribution, also confirmed the proposed mechanism of this imaging probe, as spleen is a major peripheral reservoir for CD11b^+^ cells and a resource for quick response to body inflammation^28^,^29^. Besides, serum lipid and cytokine analyses revealed obvious higher whole-body inflammatory status in ApoE^−/−^ mice as lipid profiles (TG, TC, VLDL, LDL) and classical pro-inflammatory cytokines (IL-1α, IL-10 and TNF-α) were significantly increased in ApoE^−/−^ mice, compared to C57 mice, especially the IL-1α, which is thought to be a central player in the cytokine response network that contributes to inflammation^30^. Of interest was the decreased serum IL-5 in ApoE^−/−^ mice. IL-5 has been demonstrated to reduce atherosclerosis via stimulation of anti-oxidized LDL antibody production and consequent removal of oxidized LDL from the circulation^31^,^32^. Thus, the reduction of IL-5 in the serum of ApoE^−/−^ mice might relate to increased oxidized LDL in the circulation, as oxidized LDL cholesterol is known as the driving force in atheroma formation.

It’s well known that antibodies have the intrinsic benefit of high binding affinity and high target selectivity^33^. As shown in this study, the saturation binding experiment showed that the radiolabeled anti-CD11b bound with high affinity to the Raw-264.7 cells with a K_D_ value of 14.1 ± 5.06 nM. The quick blood-pool clearance rate (blood half-life: 4.57 ± 0.59 min) contributed to a high lesion contrast with a P/B ratio being identified to be as high as 14.7 ± 2.26 at site of aortic arch and 17.3 ± 3.04 at branch point of renal artery (Fig. 6K). Moreover, the favorable *in vitro* stability of the probe that could maintain at least to 6 h after synthesis and the satisfactory half-life of the nuclide used (^99m^Tc: T_1/2_, 6.02 h) also make its clinical translation feasible in future, together with its biological origination that contributes to good biocompatibility and achievable homogenicity with the recipient^34^. However, the undesired probe distribution in liver, to some extent, impeded the visualization and assessment of atherosclerotic lesions that close to liver, which would last to 6 h post injection. Fortunately, the radioactivity accumulated in liver showed decease at 12 h post injection.

In the majority of recent-year studies regarding nuclear medicine molecular imaging studies on inflammatory cells in atherosclerosis, monocytes/macrophages were most commonly selected as targets. For instance, ^125^I/^99m^Tc-MCP-1, ^111^In-monocytes, and ^11^C/^3^H-PK11195 were fabricated to imaging infiltration of monocytes/macrophages^5^,^7^,^8^,^9^,^10^, and ^99m^Tc-Annexin V was developed to targeting their apoptosis^11^,^13^. However, inflammatory cells infiltrated in atherosclerotic plaques are not limited to monocytes/macrophages. It is reported that, about 11.8%, 0.7% and 10.8% of the total cells infiltrated were neutrophils, lymphocytes and other inflammatory cells, respectively, other than monocytes/macrophages^14^. Likewise, neutrophils and lymphocytes were respectively identified accounting about 4.3% and 6.1% of cells infiltrated in lesions of aortic aneurysms, which shared similar inflammatory process with atherosclerosis^35^. But these cells were all CD11b positive^14^,^35^. This means the previously proposed nuclear imaging probe that merely targeting monocytes/macrophages of inflamed aortic lesions might had led to a signal loss of about 10–23% compared to CD11b targeting imaging. This phenomenon may be even worse for those imaging techniques that only targeting monocytes/macrophages in apoptotic status, such as the ^99m^Tc-Annexin V^11^,^13^. However, experiment that designed to directly compare the efficacy between CD11b-targeting imaging and monocytes/macrophages targeting imaging in identifying inflamed atheroma is necessitated to confirm this speculation.

Previous study performed by von zur Muhlen *et al*., with regard to *in vivo* targeting ability of CD11b for imaging inflamed atherosclerotic plaques, advocated that imaging monocytes with SPIO nanoparticles targeting towards the monocyte integrin Mac-1 (CD11b/CD18) could not result in improved atherosclerotic plaque detection by *in vivo* MRI^36^. However, the relatively larger size of nanoparticles they used (SPIO, 113 ± 13 nm) that were prone to be captured by reticuloendothelial system^37^, and the relative lower sensitivity of MRI (compared to nuclear medicine) they used as imaging modality^38^, all posed possible reasons for the negative result of their study. Actually, a significant improved uptake of CD11b-SPIOs into monocytes/macrophages *in vitro* and a trend (although not statistically significant) towards an increased uptake of CD11b-SPIOs into atherosclerotic lesion *in vivo* compared with control-SPIO had already been observed in their study^36^. Perhaps, a particle with relatively smaller size, such as ultra-small SIPO (USPIO, <40 nm), may be more suitable for their study, as CD11b seems mainly mediate the uptake of smaller particles (<30 nm)^23^,^39^. According to von zur Muhlen’s study^36^, it at least indicated the feasibility of CD11b to be targeted to image inflamed atheroma *in vivo* if a CD11b targeting probe with smaller size were synthesized and an imaging technique with higher sensitivity were used.

In conclusion, this study demonstrates the feasibility of anti-CD11b antibody facilitated noninvasive SPECT/CT imaging of inflammatory leukocytes in murine atherosclerotic plaques. This imaging system is more sensitive to detect inflammatory cells and may present stronger signal intensity of inflamed atherosclerotic plaques compared to previous monocytes/macrophages targeting nuclear medicine imaging. By virtue of this imaging strategy, noninvasive SPECT/CT can be used to identify inflammation-rich plaques at risk for rupture and evaluate the effectiveness of inflammation-targeted therapies in atheroma.

## Methods

### Approvals

All experiments were performed following the relevant guidelines and regulations of Fudan University. This study was approved by the medical ethics committee of Zhongshan Hospital, Fudan University.

### ^99m^Tc labeling

Anti-CD11b antibody (130 kDa, BD Bioscience, New Jersey, USA), reacting with the α[M] chain of Mac-1 (CD11b/CD18, α[M]β2 integrin), was selected as targeting molecule. N-hydroxysuccinimidyl S-acetylmercaptoacetyltriglycinate (NHS-MAG_3_) was synthesized in house and then conjugated to anti-CD11b antibody following the established protocol^40^.

MAG_3_-anti-CD11b was then labeled with ^99m^Tc following the developed method (Fig. 1A)^41^. Briefly, 100 μg MAG_3_-anti-CD11b conjugate was added to combined solution of 45 μL ammonium acetate (0.25 M) and 15 μL tartrate buffer, and then no more than 25 μL (around 1 mCi) ^99m^Tc-pertechnetate generator eluate was added. Immediately after vortexing, 3 μL freshly prepared 1 mg/mL SnCl_2_∙2 H_2_O solution was added. The combined solution was incubated at room temperature for 1 h under vortexing. The labeling rate was measured by radio-TLC (Bioscan, Washington, DC, USA) using 1) thin-layer paper plate with citrate buffer (pH = 5.0) as mobile phase: ^99m^Tc-MAG_3_-anti-CD11b & colloidal ^99m^Tc, R_f_ = 0; ^99m^TcO_4_^—^, R_f_ = 0.8; 2) thin-layer silicon plate with 50% acetonitrile as mobile phase: colloidal ^99m^Tc, R_f_ = 0; ^99m^Tc-MAG_3_-anti-CD11b & ^99m^TcO_4_^—^, R_f_ = 0.7. The radiochemical purity of ^99m^Tc-MAG_3_-anti-CD11b = 1 − colloidal ^99m^Tc (%) − ^99m^TcO_4_^—^ (%). The *in vitro* stability in PBS (0.01 M), BSA solution and DMEM medium were evaluated up to 6 h at 25 °C. Same process was performed to label ^99m^Tc to isotype IgG (150 kDa, Santa Cruz Biotechnology, Inc., Texas, USA) for contrast.

### Blood kinetics and biodistribution of ^99m^Tc-MAG_3_-anti-CD11b

^99m^Tc-MAG_3_-anti-CD11b was injected via tail vein, into 5 C57 mice (male, 8w) to measure blood kinetics and into 15 C57 mice (male, 8w) to investigate biodistribution *in vivo*, with a dose of 50 μg/150μCi/mouse. The blood half-life of probe was determined with serial blood collections through tail vein after injection. For biodistribution study, 5 mice per group were killed and organs were harvested at 3, 6 and 12 hours post injection. Each sample was weighed and counts were recorded with a γ counter (CRC-15 R, Capintec Inc., Ramsey, NJ). After corrected for radioactive decay, data were calculated as percent injected dose per gram tissue (ID%/g). The mean blood half-life of ^99m^Tc-MAG_3_-anti-CD11b was estimated by monoexponential decay via GraphPad Prism 6.01 (GraphPad Software Inc.).

### Cell binding assay

The Raw-264.7 cell line was selected to test cellular affinity of ^99m^Tc-MAG_3_-anti-CD11b, while HUVEC was chose as control. All cells were cultured at 37  °C in a humidified atmosphere with 5% CO_2_. Medium was supplemented with 10% fetal calf serum and 1% antibiotic mixture (100 units/mL penicillin and 0.1 mg/mL streptomycin).

For saturation binding experiments, Raw-264.7 cells were seeded in 24-well plate with 1 × 10^6^ cells/well and cultivated overnight. Then, a concentration gradient of probe (2–80 μCi, 3–123 nM) were added to wells and incubated with cells at 37 °C for 2 h. The total volume of each well was 0.5 mL. Nonspecific binding was performed by adding same amount of probes to each well without cells at different concentrations. Cells were rinsed twice with PBS and the supernatant were removed. Then, cells of each well were collected for γ-counting. Same process was conducted to incubate Raw-264.7 with ^99m^Tc-MAG_3_-IgG and to incubate HUVEC with ^99m^Tc-MAG_3_-anti-CD11b. For blocking experiments, Raw-264.7 cells were incubated with 50-fold and 100-fold excess unlabeled antibody 2 h prior to incubation with ^99m^Tc-MAG_3_-anti-CD11b. All experimental conditions were triplicated. After each cellular experiment, the 24-well plates were imaged by BSGI to offer an overall vision of cellular affinity.

### Animal model preparation

C57 and ApoE^−/−^ mice (male, 8w) were purchased from Peking University Laboratory Animal Center (Beijing, China). The ApoE^−/−^ mice were fed with a high-fat diet (containing 21% fat and 0.15% cholesterol by weight) for more than 20 weeks to develop atheroma, while the C57 mice consumed normal chow diet containing 4% to 6% fat and a cholesterol content <0.02% (w/w). All mice were housed in a pathogen-free environment of a 12-hour light/dark cycle with free access to food and water.

### Near-infrared *ex vivo* fluorescent imaging of aortas

In order to investigate targeting ability of the antibody in tissue condition, we first performed near-infrared fluorescent imaging on excised aortas. Briefly, anti-CD11b-PE-Cy7 (BD Bioscience) was injected to 5 ApoE^−/−^ and 5 C57 mice via tail vein (1 mg/kg), while IgG-PE-Cy7 (BD Bioscience) with same dose was injected to another 5 ApoE^−/−^ mice. Twenty four hours later, mice were sacrificed. NaCl (0.9%, 5 mL) and paraformaldehyde (4%, 5 mL) were sequentially injected into vascular system to eject blood and fix vessels. Then, aorta was carefully dissected, placed on a microscope slide, covered with gauze (rinsed with 0.01 M PBS), and imaged within 1 h in the Carestream FX PRO (Kodak, USA). The Cy7 label was excited using a wavelength filter of 615–665 nm, while the emission was recorded using a 695–770 nm filter. Fluorescent images were acquired with identical exposure time for all aortas. Area percentage of plaque was measured and total photon count was recorded around plaque areas. Mean signal intensity was calculated by dividing the total photon count to the total area of plaques (SI-mean). Likewise, noise intensity (NI-mean) was measured by drawing a ROI in normal area of the aorta. Finally, a signal-to-noise ratio (SNR) was calculated as: SI-mean/NI-mean.

### Serological biochemical analysis

Before sacrificing the mice for *ex vivo* aortic fluorescent imaging, fresh blood samples were collected through left-ventricle puncture (Fig. 3). After coagulating for 20 min under house temperature, blood were centrifuged (3000 rpm × 10 min) to extract supernatant serum. Serological concentrations of TG, TC, HDL, LDL and VLDL were analyzed using an automated analyzer (Falcor 350, Menarini Diagnostics, Florence, Italy). Enzyme-linked immunosorbent assay (Elisa) kits were used to test serological IL-1α, IL-5, IL-10, IFN-γ and TNF-α according to manufacturer’s protocols, using microplate reader for Elisa (Denley Dragon Wellscan MK 3, Thermo, Finland).

### Micro-SPECT/CT imaging and imaging analysis

Micro-SPECT/CT scanning were conducted on the Nano SPECT/CT scanner (Bioscan, Washington DC, USA). ^99m^Tc-MAG_3_-anti-CD11b (1 mCi/100 μg/mouse) was injected to 5 ApoE^−/−^ and 5 C57 mice via tail vein, while ^99m^Tc-MAG_3_-IgG of same dose was injected to another 5 ApoE^−/−^ mice. Two hours later, mice were anesthetized via 2% isoflurane inhalation. CT was performed first with the following parameters: frame resolution, 256 × 512; tube voltage, 45 kVp; current, 0.15 mA; and exposure time, 500 ms/frame. Each scan took about 7 min. SPECT was performed after CT scanning with same bed position using the following parameters: four high-resolution conical collimators with 9-pinhole plates; energy peak, 140 keV; window width, 10%; resolution, 1 mm/pixel; matrix, 256 × 256; and scan time, 35 s/projection, 24 projections in all. Each mouse took 21 minutes on average. Three-dimensional ordered subset expectation maximization images were reconstructed using HiSPECT algorithm.

Reconstructed SPECT/CT data were transferred to software InVivoScope (Version 1.43, Bioscan, Washington DC, USA) for post processing. ROIs were drawn in aortic arch and at branch point of renal artery where presented obvious radioactivity (Fig. 6A). Another ROI with identical diameter (8 mm) was drawn in area of surrounding waist muscle for measuring background activity. Concentration of radioactivity (μCi/mm^3^) was automatically generated by the software for each ROI. Plaque signal was divided by background signal to yield a normalized signal intensity (P/B ratio) for inter-mice comparison.

### Flow cytometry

After *in vivo* micro-SPECT/CT imaging, each mouse was anesthetized. Fresh blood were collected through left-ventricle puncture with a 1 mL syringe which had been moistened with heparin sodium (>1000 U/mL). Red cells were lysed by lysing buffer (BD Bioscience) and removed according to manufacturer’s instruction. White cells collected were stained with anti-CD11b-PE-Cy7 (BD Bioscience) and prepared for flow cytometry test using a dual-color flow cytometer (FACS Aria II, BD Biosciences). Data were analyzed using FlowJo (Version 7.6.2 Tree Star, Inc.). The percentage of CD11b^+^ cells and the mean fluorescent intensity of CD11b^+^ cells were calculated to evaluate mobilization of CD11b^+^ cells and their expression of CD11b.

### *Ex vivo* planar imaging of aortas

In order to further eliminate potential confounding factors such as mismatch or low positive value caused by low-signal intensity, BSGI was used for *ex vivo* planar imaging of dissected aortas right after SPECT/CT imaging with following parameters: high-resolution collimator; peak energy, 140 keV; window width, 10%; resolution, 0.32 mm/pixel; matrix, 80 × 80. The collection time was 15–20 min with total counts of 80000–100000.

### Oil-Red-O staining of aorta and data analysis

Aortas from BSGI imaging (5 ApoE^−/−^ and 3 C57) and fluorescent imaging (5 ApoE^−/−^ and 3 C57) were selected for Oil-Red-O (Sigma) staining to evaluate lipid deposits inside atherosclerotic lesions (Fig. 3). The dissected aortas were opened longitudinally and fixed in 10% formalin for 5–10 min. The fixed aortas were rinsed with 60% propylene glycol for 10 min and then stained in 0.5% Oil-Red-O solution (in propylene glycol) at 37 °C for 30 min. Finally, the aortas were differentiated in a 60% propylene glycol solution for 5 min. Lipids were stained red. The stained aortas were spread on a black charpie for photographing using a digital camera under identical light condition with same photographing parameters as much as possible. Pictures were loaded to Image Pro Plus (Version 6.0, Media Cybernetics, Washington DC, USA). Area-percentages of plaques were measured, excluding all aortic branches.

### Histological Evaluation

Five aortas from ApoE^−/−^ mice and 2 from C57 mice that experienced *in vivo* SPECT/CT imaging, as well as 5 aortas from ApoE^−/−^ mice and 2 from C57 mice that experienced *ex vivo* fluorescent imaging were selected for histological evaluation (Fig. 3). Briefly, aortas were fixed, dehydrated, embedded in paraffin and sliced at aortic arch that presented obvious plaque, as well as at level of renal artery. Serial sections (5 μm) were prepared for Hematoxylin-Eosin (HE) staining and CD11b-staining. On adjacent sections, immunofluorescence staining was performed to analyze CD11b-distribution on CD11b^+^ cells with the fluorescence labelling the second antibody being Alexa Fluor 488 (BD Biosciences). The slides were also coverslipped with 4′, 6-diamidino-2-phenylindole (DAPI) to stain nucleus (Biohao Biotec Co., Ltd.). An Olympus BX35 fluorescence microscope equipped with a Nikon DS-Fil camera (Japan) was used for microscopy and image capturing. Immunohistochemical pictures captured (100× ) were analyzed with Image Pro Plus, and integrated optical density (IOD) of plaques were measured to evaluate the tissue expression of CD11b.

### Statistical analysis

We used SPSS 20.0 (IBM, Chicago, IL, USA) and GraphPad Prism 6.0 (GraphPad Software Inc.) for statistical analysis. Results were expressed as mean ± SEM. Differences between groups were evaluated by Student t test. The Tukey’s method was used to adjust *P* values for post-hoc multiple comparison after one-way ANOVA analysis. All hypothesis tests were two sided with a *P*-value < 0.05 being taken as indicating statistical significance.

## Additional Information

**How to cite this article**: Liu, G. *et al.*^99m^Tc-labelled anti-CD11b SPECT/CT imaging allows detection of plaque destabilization tightly linked to inflammation. *Sci. Rep.* **6**, 20900; doi: 10.1038/srep20900 (2016).
